# Technologies for Prediction of Preeclampsia

**DOI:** 10.17691/stm2020.12.5.09

**Published:** 2020-10-28

**Authors:** E.A. Rokоtyanskаya, I.A. Panova, A.I. Malyshkina, I.N. Fetisova, N.S. Fetisov, N.V. Kharlamova, M.V. Kuligina

**Affiliations:** Associate Professor, Department of Obstetrics and Gynecology, Neonatology, Anesthesiology, and Reanimatology; Ivanovo Research Institute of Motherhood and Childhood named after V.N. Gorodkov, Ministry of Health of the Russian Federation, 20 Pobeda St., Ivanovo, 153045, Russia; Associate Professor, Head of the Department of Obstetrics and Gynecology; Ivanovo Research Institute of Motherhood and Childhood named after V.N. Gorodkov, Ministry of Health of the Russian Federation, 20 Pobeda St., Ivanovo, 153045, Russia; Professor, Director; Ivanovo Research Institute of Motherhood and Childhood named after V.N. Gorodkov, Ministry of Health of the Russian Federation, 20 Pobeda St., Ivanovo, 153045, Russia; Associate Professor, Leading Researcher, Laboratory of Clinical Biochemistry and Genetics; Ivanovo Research Institute of Motherhood and Childhood named after V.N. Gorodkov, Ministry of Health of the Russian Federation, 20 Pobeda St., Ivanovo, 153045, Russia; Junior Researcher, Laboratory of Clinical Biochemistry and Genetics; Ivanovo Research Institute of Motherhood and Childhood named after V.N. Gorodkov, Ministry of Health of the Russian Federation, 20 Pobeda St., Ivanovo, 153045, Russia; Head of the Department of Neonatology and Clinical Pediatric Neurology; Ivanovo Research Institute of Motherhood and Childhood named after V.N. Gorodkov, Ministry of Health of the Russian Federation, 20 Pobeda St., Ivanovo, 153045, Russia; Leading Researcher, Department of Medical and Social Research, Monitoring and Supervision Ivanovo Research Institute of Motherhood and Childhood named after V.N. Gorodkov, Ministry of Health of the Russian Federation, 20 Pobeda St., Ivanovo, 153045, Russia

**Keywords:** preeclampsia, arterial hypertension, genetic polymorphisms, prediction of preeclampsia, pregnancy complication

## Abstract

**The aim of the study** was to develop technologies for predicting the development of preeclampsia (PE) based on biomedical and molecular-genetic predictors and the calculation of individual risks for this pregnancy complication.

**Materials and Methods.:**

The study involved 457 pregnant women. Of them, 147 women had chronic arterial hypertension (CAH); 109 pregnant women had CAH and secondary preeclampsia (PE); 201 patients had PE. The control group consisted of 105 pregnant women without hypertensive disorders or proteinuria. We performed a retrospective analysis of gestation course and labor outcomes, calculated risk factors using the Open Epi system and logistic regression method. Polymorphisms of genes controlling the vascular tone were identified in venous blood.

**Results.:**

There were identified risk factors for developing PE, including those in women with CAH: chronic pyelonephritis; baseline mean AP above 95 mm Hg and diastolic AP above 80 mm Hg; body mass index over 30; family history of arterial hypertension. The following were identified as additional predictors of PE: perinatal loss; premature labor; spontaneous miscarriage; PE and closed craniocerebral injuries in the past medical history; threatening miscarriage in the first trimester. Additional risk factors for PE in women with CAH were found: lack of regular antihypertensive therapy before pregnancy and in the first trimester; chronic gastritis; first pregnancy; tobacco smoking.

Polymorphic variants of the *NOS3* (-786)C allele in the genotype in combination with the heterozygous genotype in the *AGTR2* 1675G/A gene are associated with a high risk of CAH. The presence of alleles *NOS3* (-786)T/C and *NOS3* (-786)C, as well as a combination of alleles *NOS3* (-786)C and *NOS3* 894G/T, is associated with PE. The presence of alleles *AGT* 704C, *CYP11B2* (-344)T, and *GNB3* 825T/T in the genotype, both individually and in combination, is a risk factor for the development of PE secondary to CAH. The data obtained made it possible to develop a method for predicting the onset of PE in women with CAH and a model for calculating the individual risk of PE, which formed the basis for a computer program.

**Conclusion.:**

Calculating the individual risks of PE using the technologies proposed by the authors allows identifying pregnant women belonging to the high-risk group on a timely basis, which ensures high-quality implementation of preventive measures, provides a personalized approach and the possibility to prove the need for additional examination of this category of patients.

## Introduction

Preeclampsia (PE) continues to be one of the main causes of perinatal morbidity and mortality, despite the careful attention of scientists. This problem has expanded beyond the medical limits to acquire a national scale due to the high risk of complications and prevalence among the population. According to WHO, hypertensive syndrome complicates pregnancy in 4–8% of women; about 5 out of 1000 patients develop severe PE; eclampsia is diagnosed in 5 out of 10,000 [1]. PE steadily occupies the 4^th^ place among the main causes of obstetric tragedies in the Russian Federation [2]. For example, in 2018 the incidence of arterial hypertension in pregnant women was 46.9 cases per 1000 deliveries, moderate PE — 27.4 cases, severe PE — 8.4, and eclampsia — 0.12 cases [2].

A combination of many factors underlies the pathogenesis of PE. So far, there have been several different theories on the development of this pregnancy complication, therefore, prediction and prevention of PE are still far from perfect [3, 4]. Practitioners are offered to use a number of possible PE predictors that can be applied to predict the development of this pathology, both separately and in combination. However, informational characteristics and predictive value of the proposed markers are rather variable, and percentage of false positive results is high [3]. Search for the single most significant risk factor for PE has showed ineffectiveness due to the polyetiological nature of this gestational complication, which determines the use of an integrated approach to the mathematical processing of a number of the revealed PE predictors [5]. According to the literature [6, 7], the accuracy of predicting PE, especially in the first trimester of pregnancy, varies from 46.7 to 100% with reference to the existing mathematical models. Russian scientists [8] have analyzed foreign screening programs for predicting PE in the first trimester of pregnancy. Some methods have shown high predictive accuracy, however, a number of the proposed methods should be adapted prior to their application in domestic practice.

Considering the above, it seems relevant to explore modern methods of early PE prediction in order to take preventive measures on a timely basis and administer in-depth examination to women at high risk of developing this complication [9, 10]. Various kinds of information about etiological factors, pathogenetic mechanisms, and predictors of PE determine the development of new methodological approaches to processing of various data available [11]. Currently, Russian researchers propose the following mathematical models for calculating the individual risk of developing PE: a model based on the analysis of clinical and anamnestic factors and blood copeptin index measured at 11–13 weeks of gestation (sensitivity — 77%) [12]; a model of forming a risk group for PE development, obtained using logistic regression method with account of data from the past medical history (sensitivity — 75%) [13].

Genetic theory of PE pathogenesis is one of the most important. It has been proven that daughters and granddaughters of women whose pregnancy was complicated by PE are often at risk of developing this gestational complication [14]. There is evidence that about 100 gene polymorphisms are associated with PE development: detoxification genes; hemostasis systems; genes responsible for regulation of endothelial function, control of vascular tone; others [14, 15]. The risk of developing PE was found to be increased in women whose genotype contained negative polymorphisms in angiotensinogen gene, aldosterone synthase, guanine-binding protein, endothelial nitric oxide synthase, as well as associations of some polymorphic variants. However, these results are controversial; in some cases, the fact of patient’s having chronic arterial hypertension (CAH) is not taken into account [14–18].

The combination of several phenotypic and genetic factors does not always determine the development of PE in each specific case; this complication of pregnancy occurs even without the presence of well-known predictors, which makes it very difficult to predict and prevent [15, 19].

**The aim of the study** was to develop technologies for predicting the development of preeclampsia based on biomedical and molecular-genetic predictors and followed by the calculation of individual risks for this complication of pregnancy.

## Materials and Methods

A total of 457 pregnant women at 22–36 weeks of gestation were examined and divided into the following groups: group 1 included 147 women with CAH (ICD-X code О10.0), group 2 consisted of 109 women with CAH and secondary PE (ICD-X code О11), group 3 included 201 women with PE (ICD-X code О14.0). The control group consisted of 105 women without hypertensive disorders or proteinuria during pregnancy, labor, and the postpartum period. Exclusion criteria: secondary (symptomatic) arterial hypertension.

The study complies with the Declaration of Helsinki (2013) and was approved by the Ethics Committee of Ivanovo Research Institute of Motherhood and Childhood named after V.N. Gorodkov. We performed a retrospective analysis of gestation course and labor outcomes by copying data from individual case report forms of pregnant and lying-in women and labor case histories.

Peripheral venous blood was the material for the study. Molecular-genetic testing was carried out using the Proba-Rapid-Genetics reagents (DNA-technology, Russia).

Polymorphisms of genes controlling vascular tone were identified by real-time polymerase chain reaction using the “Cardiogenetics. Hypertension” kit (DNA-technology, Russia): *ADD1* G1378T (rs4961), *AGT* T704C (rs699), *AGT* C521T (rs4762), *AGTR1* A1166C (rs5186), *AGTR2* G1675A (rs1403543), *CYP11B2* C(-344)T (rs1799998), *GNB3* C825T (rs5443), *NOS3* T(-786)C (rs2070744), *NOS3* G894T (rs1799983).

**Statistical data processing.** Statistical analysis was carried out using methods of descriptive statistics in Microsoft Office 2010, Statistica 6.0 software packages for Windows and SPSS Statistics. The sample was tested for normality of distribution using the Shapiro–Wilk test. If the distribution differed from normal, the non-parametric Mann–Whitney test was used; the differences were considered statistically significant at p<0.05. To assess the significance of qualitative feature distribution between groups, two-tailed Fisher exact test was used. The relative risk (RR) was determined using the Open Epi system (http://www.openepi.com) with the calculation of a 95% confidence interval (95% CI). Logistic regression was used to find the individual contribution of each risk factor to the development of pregnancy complications. The probability of developing PE was calculated using the following formula:


$$\rho =1/(1+e^{-z}),$$

where ρ is the probability value; *е* — the base of the natural logarithm (*е*=2.72); *z*=β_1_*X*_1_+β_2_*X*_2_+…+β*_n_X_n_*+α; β — regression coefficients; α is a constant; *Х* — values of independent variables.

The quality of the obtained model for predicting PE development was assessed by calculating the area under the ROC curve — AUC (area under the curve) — by using ROC analysis.

## Results and Discussion

Analysis of clinical and anamnestic data showed that the patients included in the study were in the age ranging from 18 to 45 years; at the same time, the average age of pregnant women with CAH, including those with PE, was statistically significantly higher than in the control group and in women with only PE (p<0.05 in all cases). Nicotine dependence was more often observed in the group with CAH and secondary PE than in women of the control group (p<0.05) and patients with CAH, the relative risk of developing PE was 1.75 (95% CI 1.21–2.51; p<0.05). Extragenital pathology was statistically significantly more frequent in groups with hypertensive disorders than in the control group (p<0.05 in all cases). For example, chronic pyelonephritis increased the risk of developing PE (RR 1.39; 95% CI 1.20–1.61) and its development in women with CAH (RR 1.6; 95% CI 1.22–2.11). Chronic gastritis and gastroduodenitis were more often observed in pregnant women with CAH and secondary PE in comparison with controls and patients with CAH (p<0.05 in all cases), which increased the relative risk of developing PE (RR 1.46; 95% CI 1.09–1.96). Obesity on registration at an antenatal clinic was observed in pregnant women with hypertensive disorders more often than in the control group (p<0.05 in all cases), mainly in women with CAH, which, according to our data, increased the risk of PE (RR 1.33; 95% CI 1.13–1.56; p<0.05). A history of closed craniocerebral trauma is one of PE predictors (RR 1.26; 95% CI 1.04–1.53). It was statistically significantly more frequent in pregnant women with PE than in the control group (p<0.05) and in CAH group (p<0.05). Genetic susceptibility to arterial hypertension increased the risk of developing PE (RR 1.21; 95% CI 1.03–1.42; p<0.05), also increasing the probability of its development in women with CAH (RR 1.33; 95% CI 1.01–1.83; p<0.05). PE in previous pregnancies was more common in PE group, including PE associated with CAH, which increased the risk of developing PE in patients with CAH (RR 1.45; 95% CI 1.09–1.92; p<0.05) and without it (RR 1.52; 95% CI 1.34–1.72; p<0.05). When analyzing the obstetric and gynecological status, it was revealed that women with various types of arterial hypertension had a history of perinatal losses, spontaneous miscarriages, and preterm delivery more often as compared to the control group, which statistically significantly increased the risk of developing PE (RR 1.25; 95% CI 1.04–1.51; RR 1.40; 95% CI 1.14–1.72 and RR 1.32; 95% CI 1.09–1.59, respectively; p<0.05 in all cases). In contrast to CAH group, patients with CAH and associated PE had their first pregnancy in most cases, which increased the risk of PE development (RR 1.34; 95% CI 1.01–1.78; p<0.05). On registration, the subjects of this group had higher values of body mass index (BMI) and mean AP than women in the control group: mean AP was higher than 95 mm Hg and diastolic blood pressure was above 80 mm Hg (p<0.05 in all cases), which increased the risk of developing PE (RR 1.49; 95% CI 1.30–1.71 and RR 1.41; 95% CI 1.21–1.65, respectively). The maximum frequency of these features was observed in the group with CAH and secondary PE (p<0.05 in all cases), which increased the risk of PE development (RR 1.37; 95% CI 1.0–1.86 and RR 3.18; 95% CI 1.50–6.74, respectively), according to our data. Lack of regular intake of antihypertensive drugs at the pregravid stage and in the first trimester of pregnancy was more often observed in women with CAH and secondary PE than in those with CAH (p<0.05) and was associated with an increased risk of PE (RR 1.45; 95% CI 1.09–1.92). In women with hypertensive disorders, complications of pregnancy were statistically significantly more frequent than in patients of the control group (p<0.05): for example, the threat of miscarriage in the first trimester increased the risk of PE development (RR 1.32; 95% CI 1.13–1.54; p<0.05).

Thus, we identified PE predictors regardless of CAH presence in a woman: BMI over 30; mean AP 95 and diastolic AP over 80 mm Hg on registration; a family history of arterial hypertension; chronic pyelonephritis in the past medical history. The following were identified as additional risk factors for PE: PE, closed craniocerebral injuries; spontaneous miscarriage, perinatal loss, preterm delivery in the past history, as well as threat of miscarriage in the first trimester of the present pregnancy. The following markers of PE development were revealed in patients with CAH: lack of regular antihypertensive therapy before pregnancy and in the first trimester, first pregnancy, tobacco smoking, chronic gastritis in the past history.

Analysis of genetic polymorphisms regulating vascular tone showed that heterozygous carriership of the *AGTR2* 1675A variant was statistically significantly more frequent in CAH group (50.0±16.6%) as compared to the control group (18.9±3.2%; RR 4.2; 95% CI 1.14–15.83; p<0.05). According to the literature [20, 21], the presence of low-functional allele 1675A in the female genotype is associated with a high risk of developing PE; expression of the *AGTR2* gene decreases, the functional activity of angiotensin II type 2 receptors declines, which contributes to the development of arterial hypertension. Allele *NOS3* (-786)C was statistically significantly more frequent in the genotype of pregnant women with PE (40.5±7.9%; RR 1.9; 95% CI 1.14–3.12; p<0.05) regardless of the presence of CAH in a woman (41.9±15.7%; RR 2.0; 95% CI 1.09–3.69; p<0.05), in comparison with the control group (26.4±6.4%). Patients with CAH and secondary PE had a heterozygous genotype for the *NOS* T(-786) C polymorphism (RR 3.5; 95% CI 1.52–8.26; p<0.05) in 65.1±38.2% of cases, pregnant women with PE — in 55.8±15.2% of cases (RR 2.4; 95% CI 1.21–4.60; p<0.05), which is statistically significantly higher than in the control group (34.5±11.0%). Heterozygous variant of *NOS* 894G/T in the group with CAH and secondary PE was observed in 48.8±21.4% of cases, which was statistically significantly more frequent than in the control group (26.0±6.2%) (RR 2.7; 95% CI 1.13–6.57; p<0.05). There are different findings on the role of unfavorable polymorphisms in the *NOS* gene in the development of hypertensive complications during pregnancy: some researchers suggest they influence the pathogenesis of hypertension [22, 23], others, on the contrary, deny these facts [24]. Undoubtedly, the *NOS* 894T and *NOS* (-786) C polymorphisms cause a decrease in NO-synthase activity reducing nitric oxide synthesis and forming endothelial dysfunction, which plays an important role in the pathogenesis of PE [14].

The *AGT* 704C polymorphic variant was statistically significantly more often detected in the genotype of women with CAH and secondary PE (60.5±33.0%) than in those with CAH only (42.9±12.2%; RR 2.03; 95% CI 1.13–3.69; p<0.05). It causes increased expression of the *AGT* gene, which promotes angiotensinogen synthesis and leads to the development of arterial hypertension [25]. The *CYP11B2* (-344)T allele was predominantly found in the genotype of pregnant women with PE, regardless of the presence of CAH, (55.3±14.9 and 61.9±34.6%, respectively), in contrast to patients with CAH only (42.7±12.1%; RR 2.17; 95% CI 1.19–3.97; p<0.05 in both cases). The correlation between the *CYP11B2* (-344)T allele and the development of hypertension during pregnancy is controversial. Its effect is likely to manifest itself in combination with other risk factors for PE [26]. Allele *GNB3* 825Т/Т was observed statistically significantly more often in the genotype of women with PE secondary to CAH than in the group with CAH (in 14.3±1.7 and 2.0±0.0% of cases, respectively; RR 7.8; 95% CI 1.09–188.8; p<0.05). Some authors attribute this to the development of endothelial dysfunction, although information on the issue is controversial [26].

Analysis of total gene polymorphisms showed that association of the *NOS3* (-786)C allele and the *AGTR2* 1675G/A genotype was found in CAH group statistically significantly more often than in the control group. In PE, it was a combination of *NOS3* (-786)T/C and *NOS3* (-786)C. In patients with PE, including those with CAH, it was a combination of the *NOS3* (-786)C allele and the *NOS3* 894G/T polymorphism. The presence of alleles *AGT* 704C, *CYP11B2* (-344)T and the *GNB3* 825T/T polymorphism, both separately and in combination, was revealed statistically significantly more often in the genotype of pregnant women with CAH and secondary PE than in those with CAH (p<0.05 in all cases).

Risk factors for PE development in pregnant women with CAH specified earlier allowed us to develop a neural network system for predicting this gestational complication [27, 28] based on the use of artificial intelligence, which takes advantage of the clinical and anamnestic factors of PE development and particular issues associated with the course of hypertension before pregnancy.

In the present study, there was developed a mathematical model for individual assessment of risks for PE in pregnant women with CAH at the first trimester of gestation by calculating the discriminant function based on the identified polymorphisms of the angiotensinogen (*AGT* 704C) and aldosterone synthase (*CYP11B2* (-344)T) genes, as well as specific anamnestic and clinical data:


$$Y=0.21X_{1}+0.18X_{2}+0.31X_{3}+0.29X_{4},$$

where *Х*_1_ is the presence of the *AGT* 704C allele in the genotype: yes — 1 score, no — 0 scores; *Х*_2_ — the presence of the *CYP11B2* (-344)T allele in the genotype: yes — 1 score, no — 0 scores; *Х*_3_ — mean AP more than 95 mm Hg measured in the first trimester of pregnancy: yes — 1 score, no — 0 scores; *Х*_4_ — lack of regular antihypertensive therapy at the pregravid stage: yes — 1 score, no — 0 scores. Y≥0.68 is assumed to indicate an increased risk of PE in women with CAH; Y<0.68 is understood as no risk of developing PE (sensitivity — 67.4%; specificity — 75.5%; accuracy — 72.0%). There was formulated “a method for predicting the risk of developing preeclampsia in women with chronic arterial hypertension” [29].

**Figure 1:**
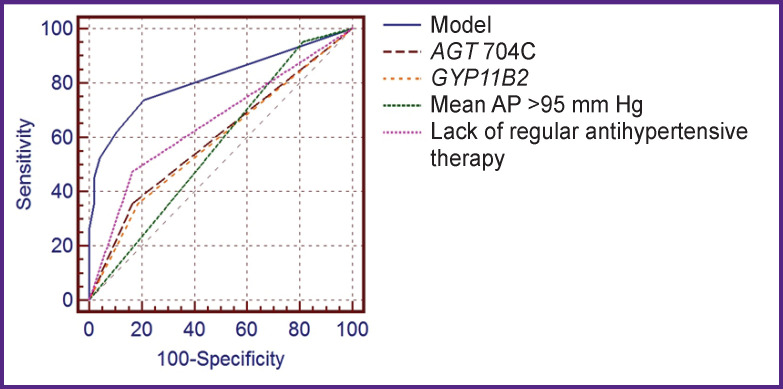
ROC-curves for predicting the risk of developing preeclampsia in women with CAH using the developed mathematical model and individual predictors in the first trimester of pregnancy

To assess the quality of the obtained model for predicting PE development in comparison with the individual contribution of each risk factor to PE development in pregnant women with CAH, we determined the area under the ROC-curve (AUC) using ROC-analysis (Figure 1). The maximum area under the ROC-curve was obtained (AUC=0.811) for PE prediction model, indicating a very good quality of the proposed model; the values of areas under the ROC-curves of individual predictors within this model were unsatisfactory and varied from 0.570 to 0.600.

Logistic regression method was used to predict the development of PE in the first trimester in women without CAH. Incremental inclusion of the identified new risk factors into the mathematical formula ρ=1/(1+*e^–z^*) made it possible to determine the most significant predictors of PE. The mathematical calculation model is as follows:


$$z=0.056+3.261X_{1}+0.976X_{2}+3.17X_{3}+1.116X_{4}+0.908X_{5},$$

where *X*_1_ is a prior history of PE; *X*_2_ — BMI >30 in the first trimester; *X*_3_ — mean blood pressure ≥95 mm Hg in the first trimester; *X*_4_ — a prior history of chronic pyelonephritis; *X*_5_ — the threat of miscarriage in the first trimester of this pregnancy. The likelihood of developing PE was determined using the 90^th^ and 10^th^ percentiles: ρ=0–0.49 — low probability; ρ=0.50–0.95 — average probability; ρ=0.96–1.0 — high probability of developing PE (sensitivity — 64.7%, specificity — 75.2% and accuracy — 68.3%).

**Figure 2:**
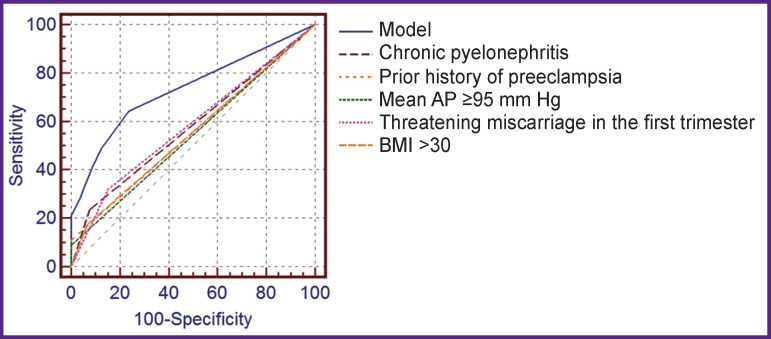
ROC-curves for predicting the risk of developing preeclampsia using the developed mathematical model and individual predictors in the first trimester of pregnancy

To analyze the quality of the obtained mathematical model for predicting PE development in comparison with the individual contribution of each risk factor to the development of this complication of pregnancy, we determined the area under the ROC-curve (AUC) (Figure 2). Only the mathematical model for predicting the development of PE has the maximum area under the curve, which corresponds to the good quality of the proposed model (AUC=0.733); the values of areas under the ROC curves of individual predictors within the model were unsatisfactory and ranged from 0.555 to 0.583.

**Figure 3:**
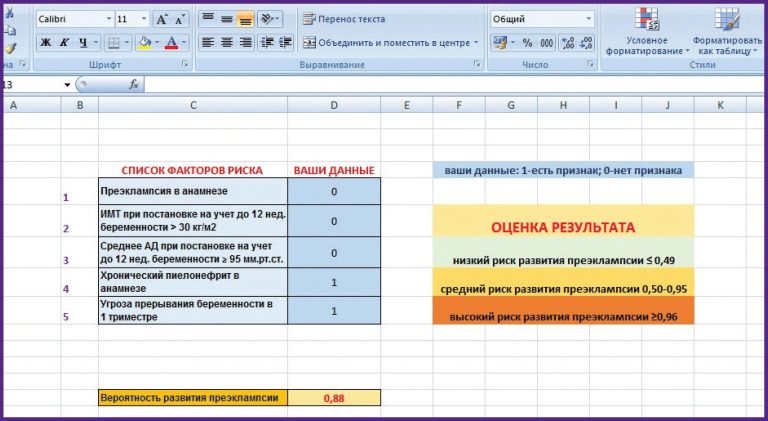
An example of forming a risk group using the calculator: average risk of developing preeclampsia

These mathematical models made it possible to develop a tool for calculating individual risk of developing PE based on Excel program (Figure 3). Digital code “0” is entered in the column “Your data” when a patient has no risk factors, “1” is entered, if there is a risk factor present. The calculation of PE development probability occurs automatically, a value from 0 to 1.0 appears in the corresponding box, next, the “Result Evaluation” column is used to predict a low, medium or high risk of developing PE.

**Figure 4:**
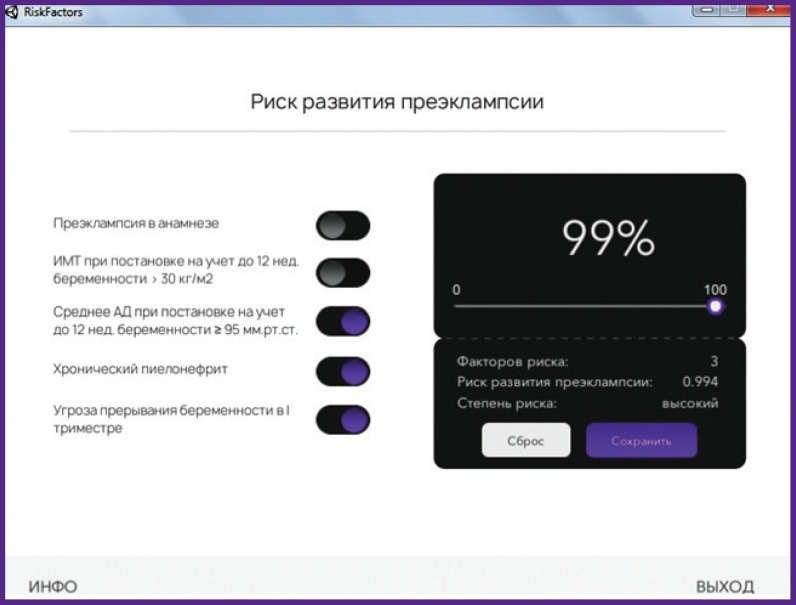
The on-screen form of the “Automated program for predicting the development of preeclampsia in pregnant women”: high risk of developing preeclampsia

The obtained mathematical model and the calculator were used to develop “Automated computer program for predicting the development of preeclampsia in pregnant women” [30]. The program is proposed for use by obstetricians and gynecologists at the outpatient stage in order to predict the risk of developing PE in women in the first trimester of pregnancy with further identification of a high-risk group and timely implementation of necessary preventive measures, as well as subsequent examination of this category of patients. The input boxes of the program interface contain information about the past medical history and examination data of the pregnant woman, entered into the indicated fields. After automatic processing, the physician receives PE risk assessment results for this patient (Figure 4).

A detailed description of instructions for working with the automated program is given in the user manual. Testing was carried out on 400 patients using sliding examination sample (see the Table).

**Table:** The results of testing the information characteristics of the “Automated program for predicting the development of preeclampsia in pregnant women”

Group	Predicted allocation to groups (absolute number/%)	
	0	1
0 (n=200)	145/72.50	55/27.50
1 (n=200)	35/17.95	165/82.50

Here: 0 — no risk of developing PE; 1 — at risk of developing PE.

The developed mathematical model showed higher characteristics than the initial ones: predictive value of the positive result was 75.0% and the negative result — 80.6%; sensitivity — 82.5%; specificity — 72.5%; accuracy — 77.5%.

Specific information on the phenotypic and molecular-genetic predictors of PE made it possible to propose a technology for predicting the development of PE in the first trimester of pregnancy. When a gynecologist collects anamnestic data of a pregnant woman at the outpatient stage, it is recommended to find out on registration before 12 weeks of pregnancy if she has a prior history of PE, chronic pyelonephritis. It is necessary to identify the signs of miscarriage in the first trimester, to calculate BMI, to measure mean AP, and to determine whether the pregnant woman belongs to the group of increased risk of developing PE using the “Automated program for predicting the development of preeclampsia in pregnant women”.

To predict the development of PE at the initial assessment of women with CAH, it is recommended to use the Neuro_Chronic neural network system for predicting preeclampsia in pregnant women with chronic arterial hypertension [27]. Apart from assessment of clinical and anamnestic data, it is necessary to determine the presence of polymorphisms of the angiotensinogen (*AGT* 704C) and aldosterone synthase (*CYP11B2* (-344)T) genes in the venous blood with subsequent data processing with the use of discriminant function. This will allow allocating the pregnant woman to the risk group for PE.

## Conclusion

The technologies developed by the authors allow calculating the individual risk of developing preeclampsia and identifying high-risk patients on a timely basis. This personalized approach ensures timely administration of preventive measures and additional examination of this category of patients, which is likely to improve perinatal outcomes.
